# Monoamine-oxidase Type B Inhibitors and Cognitive Functions in Parkinson’s Disease: Beyond the Primary Mechanism of Action

**DOI:** 10.2174/1570159X20666220905102144

**Published:** 2023-04-12

**Authors:** Domiziana Rinaldi, Marika Alborghetti, Edoardo Bianchini, Michela Sforza, Silvia Galli, Francesco E. Pontieri

**Affiliations:** 1 Dipartimento di Neuroscienze, Salute Mentale e Organi di Senso, Sapienza Università di Roma, Italy;; 2 IRCCS Fondazione Santa Lucia, Roma, Italy

**Keywords:** Cognition, Parkinson’s disease, MAO_B_ inhibitors, rasagiline, safinamide, selegiline

## Abstract

Symptoms of cognitive impairment are rather common since the early stage of Parkinson’s disease (PD); they aggravate with disease progression and may lead to dementia in a significant proportion of cases. Worsening of cognitive symptoms in PD patients depends on the progression of subcortical dopaminergic damage as well as the involvement of other brain neurotransmitter systems in cortical and subcortical regions. Beyond the negative impact on disability and quality of life, the presence and severity of cognitive symptoms may limit adjustments of dopamine replacement therapy along the disease course.

This review focuses on the consequences of the administration of monoamine-oxidase type B-inhibitors (MAO_B_-I) on cognition in PD patients. Two drugs (selegiline and rasagiline) are available for the treatment of motor symptoms of PD as monotherapy or in combination with L-DOPA or dopamine agonists in stable and fluctuating patients; a further drug (safinamide) is usable in fluctuating subjects solely.

The results of available studies indicate differential effects according to disease stage and drug features. In early, non-fluctuating patients, selegiline and rasagiline ameliorated prefrontal executive functions, similarly to other dopaminergic drugs. Benefit on some executive functions was maintained in more advanced, fluctuating patients, despite the tendency of worsening prefrontal inhibitory control activity. Interestingly, high-dose safinamide improved inhibitory control in fluctuating patients. The benefit of high-dose safinamide on prefrontal inhibitory control mechanisms may stem from its dual mechanism of action, allowing reduction of excessive glutamatergic transmission, in turn secondary to increased cortical dopaminergic input.

## 1 INTRODUCTION

### 1.1 Parkinson’s Disease

Parkinsonism refers to any clinical condition featuring bradykinesia combined with rigidity or resting tremor. Idiopathic Parkinson’s disease (PD) is the most frequent cause of parkinsonism in adults. PD is a chronic progressive neurodegenerative condition of unknown etiology characterized by degeneration of dopamine (DA)-containing neurons in the mesencephalic substantia nigra *pars compacta* [1]. Besides parkinsonism, PD patients frequently experience a variety of non-motor symptoms (NMS), including autonomic, neuropsychiatric, sleep, sensory and cognitive disturbances [2]. Some NMS may even precede the onset of motor symptoms [2, 3].

The pathophysiology of NMS in PD is rather complex. Clinical-pathological studies showed the involvement of several brainstem and cortical regions containing both dopaminergic and non-dopaminergic neurons. In particular, dopaminergic, serotonergic, noradrenergic, cholinergic, GABAergic and glutamatergic mechanisms co-participate in the expression of several NMS.

### 1.2 Cognitive Impairment in PD

Cognitive symptoms are relatively frequent in PD patients, being present in up to 83% of cases [2]. Cognitive impairment negatively influences the autonomy and quality of life of affected subjects [3, 4] and aggravates caregiver burden [2, 4]. In early PD stage, cognitive impairment takes the features of the dysexecutive syndrome, with a deficit of attention, working memory, information processing, and verbal and visual spatial functions [3, 5, 6]. These symptoms depend mostly on dopaminergic denervation in frontostriatal circuitries regulating cognitive and affective responses, in particular the dorsolateral prefrontal cortex, and less severely the orbitofrontal and anterior cingulate cortices [6-8]. Interestingly, DA replacement therapy (DRT) administered to drug-naïve and/or early-stage PD patients for controlling motor symptoms may ameliorate cognitive and affective features as well [9-13].

With disease progression, however, dysexecutive symptoms progressively worsen, and more than 20% of patients evolve into the condition of PD-dementia [7, 8] with memory impairment, disorientation, and multiple deficits of cortical functions. These cognitive symptoms are frequently associated with neuropsychiatric features, such as visual hallucinations, delusions, and psychosis. The main pathophysiological mechanism underlying dementia in PD is the spreading of alpha-synuclein aggregates (Lewy bodies) to limbic structures and neocortical regions [14]. Moreover, amyloid plaques and tau-related pathology may occur, suggesting a possible overlap with Alzheimer’s Disease [15]. In the advanced PD stage, multiple neurotransmitter/receptor systems contribute to cognitive impairment. Derangement of dopaminergic projections to prefrontal regions and the striatum is related to disturbances of cognitive flexibility, planning, problem-solving, task switching, verbal fluency, working memory and inhibitory control [16]. Cholinergic dysfunction is responsible for the impairment of selective attention and strengthening of salience [17, 18], as well as posterior cortical deficits [5, 17-19]. Impairment of adrenergic projections from the *locus coeruleus* to the prefrontal cortex compromises vigilance, responsiveness, cognitive flexibility, and inhibitory control [20, 21]. Serotonergic dysfunction has a crucial role in mood disorders, hallucinations, and psychosis [22], and contributes to motor, cognitive and neuropsychiatric features of advanced PD stage through complex interactions with GABAergic and glutamatergic pathways [22, 23]. Glutamate signaling plays an important regulatory role in neuronal activity in the prefrontal cortex by modulating working memory and some neuropsychiatric features in PD [5, 8, 24]. To this end, memantine, a partial NMDA receptor antagonist, has been shown to ameliorate attention and episodic memory [5, 25], and amantadine, a low-affinity, non-competitive NMDA receptor antagonist, seems to enhance memory performances in PD patients with dementia [26]. As a consequence of the multiple neurochemical alterations and the progression of DA denervation, DRT may aggravate cognitive and neuropsychiatric symptoms in advanced PD patients.

## 2 MONOAMINE-OXIDASE TYPE B INHIBITORS FOR PD TREATMENT

To date, L-3,5-dihydroxyphenylalanine (L-DOPA) remains the mainstay symptomatic therapy for PD. To prevent excessive peripheral metabolism and ensure adequate crossing of the blood-brain barrier, the drug is administered together with L-aromatic-aminoacid-decarboxylase (LAAD) inhibitors (carbidopa, benserazide). Despite LAAD inhibition, the plasma half-life of oral L-DOPA remains relatively short, and this contributes to producing pulsatile stimulation of striatal dopaminergic receptors.

The occurrence of fluctuations may be viewed as a milestone in PD progression. Fluctuations refer to the predictable or unpredictable worsening of symptoms of parkinsonism occurring during daytime and along nighttime [1, 3, 27], which can be observed after months/years of exposure to L-DOPA in the majority of PD patients. Progression of neuronal cell loss is the primary mechanism generating fluctuations in PD [28, 29]. In the moderate-advanced disease stage, buffering of extracellular L-DOPA and DA by residual dopaminergic neurons becomes insufficient and significant uptake of DA and L-DOPA occurs *via* serotonergic neurons and glial cells. Pulsatility of plasma L-DOPA levels following oral administration represents a further exogenous mechanism underlying fluctuations, being associated with time- and dose-dependent variations of substrate availability. Under these conditions, therefore, transmitter release is regulated by either tonic (as in the case of serotonergic neurons) or osmotic (as in the case of glial cells) mechanisms. Consequently, postsynaptic dopaminergic receptors undergo non-physiologic stimulation that activates maladaptive processes within striatal neurons, in turn causing L-DOPA-induced dyskinesia (LID) [30]. As anticipated previously, similar consequences may occur at the level of circuitries modulating non-motor functions, producing non-motor fluctuations and peak-dose worsening of some NMS due to excessive stimulation of postsynaptic dopaminergic receptors [4, 12]. To overcome significant pulsatility of dopaminergic receptor stimulation and promote continuous dopaminergic stimulation of the affected striata, attempts have been made to reduce L-DOPA metabolism at peripheral and/or central compartments. Indeed, continuous dopaminergic stimulation through L-DOPA/carbidopa intestinal gel (LCIG) or subcutaneous infusion of apomorphine has proved to have great efficacy in reducing fluctuations and dyskinesia in advanced PD patients [31, 32].

Metabolism of L-DOPA occurs through two distinct pathways, the catechol-O-methyltransferase (COMT) and type-B monoamine-oxidase (MAO_B_). The latter is an isoform of the flavin MAO family that catalyzes the oxidative deamination of monoamines, with high selectivity for DA and tyramine, in contrast to MAO_A_ that is primarily involved in the metabolism of serotonin and norepinephrine. While MAO_A_-inhibitors are used primarily for treating depression, three MAO_B_-inhibitors (MAO_B_-I) have therapeutic applications in PD: selegiline, rasagiline, and safinamide.

There are several distinctive features among these drugs, suggesting potentially different safety and efficacy profiles. Randomized controlled trials demonstrated that all these treatments are effective for motor fluctuations [33-40] in moderate-advanced PD patients. A recent work conducted by PD MED Collaborative Group investigated 500 PD patients without dementia who showed motor complications and underwent adjunct therapy with COMT or MAO_B_ inhibitors. Results showed no difference in terms of quality of life in patients treated with direct dopaminergic agonists or COMT and MAO_B_ inhibitors, but MAO_B_-I was superior for both Parkinson’s Disease Questionnaire-39 (PDQ-39) and EuroQOL-5D-3L (EQ-5D-3L) scores [41]. Selegiline and rasagiline may be administered in the early disease stage as well [42-44].

In this work, we focused on the effects of MAO_B_-I on cognitive functions in PD patients. To this end, we reviewed the literature for studies reporting the outcome of cognitive tasks in PD patients receiving MAO_B_-I. Reports were identified through the search on PubMed using a combination of the following keywords: “cognitive”, “executive functions”, “verbal fluency” AND “Parkinson’s disease” AND “selegiline” OR “rasagiline” OR “safinamide”. Papers published in the period from 1978 to 2021 were evaluated independently by two reviewers (DR and MA) for consistency. Any disagreement was addressed by further discussion with the other authors. Moreover, references from selected papers were reviewed carefully in search of further indications. The papers were selected on the basis of the year of publication, the type and number of the PD population under investigation, the type and duration of the therapy with MAO_B_-I, and the expected outcomes. These data are summarized in Table **1**.

### 2.1 Selegiline

Selegiline (L-deprenyl; (R)-(–)-N,α-dimethyl-N-(2-pro-pynyl) phenethylamine) is a selective and irreversible MAO_B_-I, which is readily absorbed from the gastrointestinal tract and promptly crosses the blood-brain-barrier. It is metabolized into the liver in desmethylselegiline and methamphetamine, which are in turn transformed into amphetamine [45]. Selegiline is currently used at daily doses of 5 or 10 mg as monotherapy or in combination with L-DOPA and/or direct dopamine agonists. Oral as well as transdermal routes of administration are available in most countries. Studies demonstrate that selegiline is safe and well-tolerated in PD patients, with the rare occurrence of side effects of MAO-inhibitors, such as sexual dysfunction and weight gain. Selegiline improves motor symptoms in PD patients and may contribute to reducing L-DOPA equivalent daily dose (LEDD) [34, 45]. Despite the initial speculation on the antioxidant action of selegiline and its potential neurotrophic effect suggested by results from the DATATOP trial [33], more recent studies have not confirmed such a hypothesis [27, 46].

The benefit of selegiline in PD may extend to some NMS as well (Table **1**). With respect to cognitive symptoms, Hietanen and collaborators (1991) performed a 4-week, double-blind, placebo-controlled, pilot trial on 18 L-DOPA-naïve PD patients [47]. A comprehensive cognitive and psychomotor battery test was performed. Despite the observation of slight improvement in a few subjects, there were no significant differences between selegiline and placebo [47]. Findings from a further 8-week, randomized, placebo-controlled, double-blind trial published in 1995 showed the improvement in a general measure of mentation/mood and UPDRS-II by selegiline, with no significant difference in a more specific cognitive test, such as the Wisconsin Card Sorting Test (WCST) or Raven’s Advanced Progressive Matrices Test (APM) [48]. Eventually, Murakami *et al.* compared the effects of several dopaminergic treatments (L-DOPA, DA agonists, selegiline) on motor and cognitive function in 27 drug-naïve PD patients. Motor (MDS-UPDRS-III), cognitive (Montreal Cognitive Assessment – MoCA) and behavioral (Neurobehavioral Cognitive Status Examination – COGNISTAT) outcome measures were used. The results showed the improvement of gait and language function in the whole cohort of patients, but there was no analysis on selegiline-treated subjects alone [49].

In more advanced patients, the effects of a 4-week add-on with selegiline to L-DOPA were investigated by Portin *et al.* (1983) by measuring changes in verbal and non-verbal memory, language functions, visuomotor speed, motor speed, and vigilance in PD subjects [50]. Authors highlighted two different patterns of response according to baseline cognitive status: subjects with progressive dementia displayed considerably more emotional and behavioral changes and failed to respond to treatment. Conversely, subjects without dementia showed a trend toward improvement in cognitive performances, in particular memory, naming and motor speed. Moreover, selegiline increased arousal and ameliorated fatigue and tiredness [50].

### 2.2 Rasagiline

Rasagiline ((R)-N-2-Propynyl-1-indanamine) is a selective and irreversible MAO_B_-I, with a therapeutic indication in PD patients as monotherapy [43, 44] or in combination with L-DOPA [35, 36]. Differently from selegiline, rasagiline is not metabolized into amphetamine-like compounds [13, 46]. Besides the efficacy of rasagiline on motor symptoms of PD, studies were carried out on the potential benefit of the drug on cognitive functions (Table **1**). In a 12-week, double-blind, placebo-controlled trial, Barone and collaborators (2015) investigated the effects of rasagiline on cognitive and depressive symptoms in non-demented PD patients co-diagnosed with depression [51]. Despite the lack of differences between rasagiline and placebo on either cognitive or affective outcomes, a post-hoc analysis revealed a significant effect in favor of rasagiline on the UPDRS-I depression domain, PDQ-39 mobility and some cognitive items.

Haganasi *et al.* investigated the effects of rasagiline on cognitive functions in non-demented PD patients. In this randomized, double-blind, placebo-controlled trial, 55 PD patients with impairment of at least 2 cognitive domains among attention, memory, executive and visual-spatial functions were randomized to rasagiline 1 mg or placebo. Rasagiline group displayed significant improvement on several neuropsychological tests, including the digit span-backward, the verbal fluency test, the attentional Z test and the Stroop Word Color Test (SWCT) [52]. The efficacy of rasagiline on executive functions in PD patients was supported by a more recent (2018) study from our group, carried out on fluctuating PD patients. Under these conditions, add-on with rasagiline 1 mg was accompanied by significant improvement in the Frontal Assessment Battery (FAB) total score at the end of the L-DOPA dose. Interestingly, add-on with rasagiline ameliorated all FAB subdomain but inhibitory controls, as if increased dopaminergic activity at the end of L-DOPA dose in fluctuating patients might somehow reduce prefrontal pathways modulating response behavior [53].

Weintraub and collaborators investigated the effects of rasagiline in PD patients diagnosed with mild cognitive impairment (MCI). In this study, 170 PD-MCI patients under stable DAergic therapy were randomized to rasagiline or placebo treatment for 24 weeks. The results did not show significant differences in any cognitive domain, despite the improvement of motor symptoms and activities of daily living in the rasagiline group [54]. Similarly, Frakey and colleagues were unable to measure any significant difference in neuropsychological functions at the end of a 6-month treatment period with rasagiline or placebo in a cohort of 50 mild-moderate, non-demented PD patients [55].

PD Parkinson’s disease; NPS Neuropsychological; ROT Rod Orientation Test; SCWT Stroop Color and Word Test; BDI Beck depression inventory; RBMT Rivermead Behavioural Memory Test; APM advanced progressive matrices; MDS-UPDRS Movement Disorder Society-Unified Parkinson’s Disease Rating Scale; MoCA Montreal Cognitive Assessment; PDQ-39 The Parkinson’s Disease Questionnaire 39; FAB Frontal Assessment Battery; DST Digit Symbol Substitution Test; TMT Trail Making Test; VFT Verbal Fluency Test; GRID-HAMD GRID-Hamilton Depression Rating Scale; NMS Non Motor Symptoms; SCOPA SCales for Outcomes in PArkinson’s disease; MMSE Mini-Mental State Examination; CISI Clinical Impression of Severity Index; HADS Hospital Anxiety and Depression Scale; PDSS The Parkinson's disease sleep scale; EQ Health Questionnaire; DSB Digit Span Backwards Test.

### 2.3 Safinamide

Safinamide ((S)-2-((4-((3-Fluorobenzyl)oxy)benzyl) amino) propanamide) is a reversible MAO_B_-I, displaying extremely high specificity for MAO_B_ [46]. Besides MAO_B_ inhibition, high-dose safinamide inhibits voltage-sensitive-sodium (VSSC_s_) and -calcium channels (N- and L-type VSCC_s_) [46]. Inhibition of L-type VSCC_s_ occurs at high concentration solely, therefore limiting the effects of safinamide on blood pressure regulation; conversely, VSSC_s_ inhibition is voltage-, use-, and frequency-dependent, and becomes relevant with repetitive neuronal firing [56]. Inhibition of VSSC_s_ rather than VSCC_s_ may affect the modulation of glutamate release *in vitro* as well as *in vivo* [57]. Indeed, safinamide restrains glutamate and GABA release in the hippocampus, inhibits depolarization-evoked glutamate release in the globus pallidus, subthalamic nucleus and substantia nigra pars reticulata, but not in the dorsal striatum, and may reduce glutamatergic overdrive of corticofugal and subthalamic efferent fibers [56, 57].

Safinamide was originally developed as an antiepileptic drug, and eventually approved by the FDA as an add-on to L-DOPA in PD patients with motor fluctuations. It is administered orally at daily doses of 50 or 100 mg and has high availability (80-92%) [58]. Unlike selegiline and rasagiline, safinamide is not metabolized by the cytochrome system, and therefore does not cause any relevant drug interaction [58]. In humans, the daily dose of 50 mg is required for reversible full inhibition of MAO_B_ activity. The daily dose of 100 mg also inhibits glutamate release, an effect that may contribute to further efficacy on motor and NMS in advanced PD patients. Moreover, the modulation of glutamatergic overactivity might contribute to limiting excitotoxicity in neurodegenerative disorders [59]. Moving to symptomatic issues, the anti-glutamatergic action of high-dose safinamide may be relevant to counteract LIDs in PD. Such hypothesis is supported by findings from Study 016 and SETTLE trials, two phase-III clinical studies showing that 24-week treatment with safinamide reduces “off”-time and improves overall clinical status and “on” time without troublesome dyskinesia in fluctuating PD patients [39, 60]. In particular, in the subgroups of patients with moderate-severe dyskinesia at baseline, safinamide 100 mg significantly reduced dyskinesia rating scale score (Study 018, the 18-month extension of study 016) [61]. Moreover, a recent, multicenter, observational, retrospective cohort study (SYNAPSES trial) investigating fluctuating PD patients for 12 months, with particular attention to subjects aged >75 years with relevant comorbidities and psychiatric conditions, confirmed the efficacy and safety profiles of safinamide [62].

As anticipated above, the positive effects on fluctuations and the anti-glutamatergic activity of high-dose safinamide may be beneficial to NMS as well (Table **1**). Safinamide 100 mg improved the emotional well-being domain of PDQ-39 and GRID-HAMD scores [63] and ameliorated painful cramps, muscle spasms and restless leg symptoms [64-66]. In a retrospective study on 20 PD patients, Bianchi and collaborators (2019) reported a statistically significant benefit on the Parkinson’s Disease Non-Motor-Symptom Scale (PD-NMSS) score after 3 months of add-on therapy with safinamide to fluctuating patients [67]. Analysis of the NMS subscores showed that the effects were more pronounced in mood/cognition and attention/memory domains [67]. Twenty fluctuating PD patients were tested by De Micco and colleagues (2022) in terms of NMS at baseline and after 6 months of treatment with safinamide 50 mg once daily. They observed stable cognitive functioning after treatment with safinamide 50 mg, speculating on the non-use of glutamatergic inhibition at the base of these results [68]. To further investigate the potential for glutamatergic inhibition by safinamide on cognitive functions, we performed recently (2021) an observational, prospective, exploratory study on 35 non-demented PD patients with motor fluctuations [69]; add-on with safinamide 100 mg improved executive functions, especially attention and inhibition of cognitive interference at the end of L-DOPA dose. These findings suggest the improvement of regulatory activity by the anterior cingulate cortex and dorsolateral PFC by high-dose safinamide in fluctuating PD subjects [69]. Thus, safinamide seems to have a beneficial impact on a great variety of NMS, which may be attributable to the modulation of glutamate transmission, the additional mechanism of action that distinguishes safinamide from other MAO_B_-I.

## CONCLUSION AND FUTURE PERSPECTIVE

The results of studies reviewed herein show that MAO_B_-I may affect cognitive functions in PD patients and suggest differential effects according to disease stage and specific pharmacological properties of each drug.

Both selegiline and rasagiline [49-53] may ameliorate dysexecutive symptoms in the early stages of PD. Data on safinamide are missing due to the availability of the drug as an add-on to L-DOPA in fluctuating subjects solely [63, 67-69]. The effects of selegiline and rasagiline in early PD patients are similar to those of other antiparkinsonian drugs [33, 34, 36, 42-44, 46, 48] and, in general, support the idea that dysexecutive symptoms at this stage primarily depend on reduced dopaminergic transmission in prefrontal cognitive circuitries; the attempts to identify drug- or class-specific effects are insufficient at present. Thus, under these conditions, the benefit of cognitive functions appears independent of the specific mechanism of action of each drug. Despite this general observation, however, there is initial evidence supporting the preferential benefit of selegiline on arousal [46, 70], an effect probably mediated through the stimulant actions of amphetamine-like metabolites of this drug. With this respect, however, the possible neurotoxic effects of amphetamine-like derivatives [45, 70, 71], determined in preclinical studies, should be taken into account.

Increased DA transmission retains a significant role in determining the consequences of MAO_B_-I on cognitive functions in moderate to advanced disease stages as well. Under these conditions, however, the interactions between iatrogenic effects and neuropathology become more complex. The study by Portin *et al.* on the effects of selegiline on cognitive status in PD patients with different baseline cognitive levels is, to our opinion, emblematic [50]. Patients without dementia displayed benefits in several cognitive domains, whereas those with signs of cognitive impairment underwent neuropsychiatric side effects and failed to respond to treatment. These results suggest that more pronounced dopaminergic denervation and spreading of neuropathology to neocortical regions make patients more prone to the negative consequences of excessive dopaminergic input to the prefrontal cortex. We believe this concept is fundamental for correct managing of the advanced stages of PD, since it poses potential boundaries to further increase DRT.

Worsening of cognitive functions is a well-known adverse event of dopaminergic therapy in subjects affected by PD-dementia [5, 7-9, 19, 25, 72, 73]. However, negative consequences on impulse control may be observed since the early disease stage, in particular following administration of direct dopamine agonists [7, 13, 74-76]. As to this issue, there is still controversy on whether behavioural changes in treated PD patients primarily depend on iatrogenic action, reduced ability of cortical neurons to manage high dopaminergic input or both [2, 9, 12, 76-80]. Findings from two recent studies from our group confirm that increased DRT in non-demented, fluctuating PD patients may already worsen prefrontal inhibitory control. Thus, add-on with rasagiline to fluctuating PD patients ameliorated total FAB score despite worsening of inhibitory control domain [53]. Under similar conditions, however, add-on with safinamide 100 mg produced similar global benefit together with significant improvement of the inhibitory control domain [69]. Interestingly, rasagiline and high-dose safinamide display similar inhibition of MAO_B_ activity [46], and comparable power for calculation of daily levodopa-equivalent dose [81-83]. Thus, the benefit of high-dose safinamide on prefrontal inhibitory control mechanisms may stem from the reduction of excessive glutamatergic transmission, in turn, secondary to increased cortical dopaminergic input. This hypothesis is further supported by the observation that the effects of safinamide 100 mg were not replicated by lower drug dose (50 mg) [46, 58], which does not affect glutamatergic transmission [46]. The effects of high-dose safinamide on cognitive functions [63, 67-69] are in line with reports from other authors on pain [64, 65, 84], urinary symptoms [68, 85], and LIDs [27, 38]. LIDs, in particular, reflect a maladaptive form of activity-dependent synaptic plasticity at excitatory corticostriatal synapses modulating firing of striatofugal direct pathway neurons and are related to increased glutamate release in the neostriatum and substantia nigra *pars reticulata* [30, 86]. The pathophysiology of LIDs strictly depends on striatal long-term-potentiation (LTP) at excitatory synaptic transmission. This mechanism requires the activation of D1 receptors together with several subtypes of glutamate receptors, including NMDS, mGlu1 and mGlu5 receptors [30, 87-89]. DA denervation makes this mechanism inflexible; thus, LIDs may persist even after withdrawal from L-DOPA [30, 90]. Abnormal cortical facilitation in the primary motor cortex may concur with the development of LIDs, as evidenced by Guerra and collaborators (2019, 2022) [91, 92]. The same authors showed that safinamide 100 mg was able to restore the underlying dysfunction thanks to its anti-glutamatergic activity, and may prevent LIDs from worsening over time [91, 92]. Similar interactions between dopaminergic and glutamatergic transmission may underlie some NMS, such as cognitive and sensory alterations. Consequently, the dual mechanism of action of high-dose safinamide may produce a stronger benefit than simple inhibition of MAO_B_ activity in complicated PD patients. Further controlled studies are needed to confirm these results in order to refine therapeutic potentials of pharmacological therapy of PD.

## Figures and Tables

**Table 1:** Effects of selegiline, rasagiline, and safinamide on cognitive domain in PD patients.

**Authors**	**Population**	**Drug**	**Time**	**Outcomes**	**Results**
Hietanen, 1991 [41]	L-DOPA-naïve PD patients	Selegiline, up to 30 mg/kg	12 weeks	NPS tests	No difference between selegiline and placebo
Dalrymple-Alford *et al.*, 1995 [48]	L-DOPA-naïve PD patients	Selegiline 10 mg/day	8 weeks	ROT, SWCT, BDI, RBMT, AMP scores, MDS-UPDRS II score	Selegiline improved measures of mentation/mood and MDS-UPDRS II
Murakami *et al.*, 2016 [49]	Drug-naïve PD patients	L-DOPA, DA agonists, Selegiline	4-7 months	MoCA-J, COGNISTAT-J scores, MDS-UPDRS score	Improvement in gait and language function, no analysis on selegiline-treated group alone
Portin and Rinne, 1983 [50]	Advanced PD patients, with or without dementia	Selegiline 10 mg/day	4 weeks	Memory, cognitive functions, vigilance, and emotional processes	Patients without dementia tended to improve in memory and motor speed
Barone *et al.*, 2015 [51]	Non demented PD patients co-diagnosed with depression	Rasagiline 1 mg/day	12 weeks	BDI, NPS tests, PDQ-39, MDS-UPDRS score	No difference between Rasagiline and placebo on depressive symptoms or cognition
Hanagasi *et al.*, 2011 [52]	Non demented PD patients, with a mild cognitive impairment	Rasagiline 1 mg/day	3 months	NPS tests	Rasagiline improved DST, VFT, Z scores, SWCT
Rinaldi *et al.*, 2018 [53]	PD patients with motor fluctuations	Rasagiline 1 mg/day	16 weeks	FAB 20 min before the second scheduled daily dose of L-DOPA	Rasagiline determined an improvement in FAB score at the end of L-DOPA dose
Weintraub *et al.*, 2016 [54]	PD patients in stable DRT and mild cognitive impairment	Rasagiline 1 mg/day	24 weeks	SCOPA-Cognition scores	No difference between Rasagiline and placebo in cognition, but improved motor symptoms
Frakey and Friedman, 2017 [55]	Non demented PD patients	Rasagiline 1 mg/day	6 months	DST, TMT, VFT scores	No difference between Rasagiline and placebo
Cattaneo *et al.*, 2017 [60]	PD patients with motor fluctuations	Safinamide 100 mg/day	2 years	PDQ-39, GRID-HAMD scores	Safinamide 100 mg improved emotional well-being
Cattaneo *et al.*, 2017 [60]	PD patients with motor fluctuations	Safinamide 100 mg/day	24 months	PDQ-39	Safinamide 100 mg had positive effect on pain
Cattaneo *et al.*, 2018 [63]	PD patients with motor fluctuations	Safinamide 100 mg/day	2 years	PDQ-39	Safinamide showed positive effects on chronic pain
Bianchi *et al.*, 2019 [67]	PD patients with motor fluctuations	Safinamide 50 mg/day and then 100 mg/day	3 months	PD-NMS scale, SCOPA, MMSE, CISI-PD, HADS, PDSS, PDQ-8, EQ-5D	Safinamide improved NMS, especially mood/cognition and attention/memory domains
De Micco *et al.*, 2022 [68]	PD patients with motor fluctuations	Safinamide 50 mg/day	6 months	Motor and non-motor evaluations	Stable cognitive functions after SF treatment
Rinaldi *et al.*, 2021 [69]	PD patients with motor fluctuations	Safinamide 100 mg/day	12 weeks	MDS-UPDRS III, FAB, SWCT	Safinamide improved executive functions, especially attention and cognitive interference

**Note:** Effects of selegiline, rasagiline and safinamide on cognitive domain and non-motor symptoms in patients with Parkinson's disease at various stages. The table describes the clinical studies carried out, specifying the first author, year of publication, study population, drug used and dosage, duration of treatment, outcomes and obtained results.

## LIST OF ABBREVIATIONS

APM: Advanced Progressive Matrices
BDI: Beck Depression Inventory
CISI: Clinical Impression of Severity Index
COGNISTAT: Neurobehavioral Cognitive Status Examination
COMT-I: Catechol-O-methyltransferase Inhibitor
DA: Dopamine
DRT: Dopamine Replacement Therapy
DSB: Digit Span Backwards Test
DST: Digit Symbol Substitution Test
EQ: Health Questionnaire
EQ-5D-3L: European Quality of Life 5 Dimensions 3 Level Version
FAB: Frontal Assessment Battery
GRID-HAMD: GRID-Hamilton Depression Rating Scale
HADS: Hospital Anxiety and Depression Scale
LAAD: L-aromatic-aminoacid-decarboxylase
L-DOPA: Levodopa
LCIG: Levodopa-carbidopa Intestinal Gel
LID: Levodopa-induced Dyskinesia
LTP: Long-term-potentiation
MAO_B_-I: Monoamine-oxidase type B-inhibitors
MCI: Mild Cognitive Impairment
MDS-UPDRS: Movement Disorder Society-Unified Parkinson’s Disease Rating Scale
MMSE: Mini-Mental State Examination
MoCA: Montreal Cognitive Assessment
NMS: Non-motor Symptoms
NMSS: Non-Motor-Symptom Scale
PD: Parkinson’s Disease
PDQ-39: The Parkinson’s Disease Questionnaire 39
PDSS: The Parkinson's Disease Sleep Scale
ROT: Rod Orientation Test
RBMT: Rivermead Behavioral Memory Test
SCOPA: SCales for Outcomes in PArkinson’s disease
SCWT: Stroop Color and Word Test
TMT: Trail Making Test
VFT: Verbal Fluency Test
WCST: Wisconsin Card Sorting Test

## References

[1] Kalia L.V., Lang A.E. (2015). Parkinson’s disease.. Lancet.

[2] Schapira A.H.V., Chaudhuri K.R., Jenner P. (2017). Non-motor features of Parkinson disease.. Nat. Rev. Neurosci..

[3] Poewe W., Seppi K., Tanner C.M., Halliday G.M., Brundin P., Volkmann J., Schrag A.E., Lang A.E. (2017). Parkinson disease.. Nat. Rev. Dis. Primers.

[4] Hiseman J.P., Fackrell R. (2017). Caregiver burden and the nonmotor symptoms of Parkinson’s disease.. Int. Rev. Neurobiol..

[5] Aarsland D., Brønnick K., Fladby T. (2011). Mild cognitive impairment in Parkinson’s disease.. Curr. Neurol. Neurosci. Rep..

[6] Kudlicka A., Clare L., Hindle J.V. (2011). Executive functions in Parkinson’s disease: Systematic review and meta-analysis.. Mov. Disord..

[7] Emre M. (2004). Dementia in Parkinson’s disease: Cause and treatment.. Curr. Opin. Neurol..

[8] Aarsland D., Creese B., Politis M., Chaudhuri K.R. (2017). ffytche, D.H.; Weintraub, D.; Ballard, C. Cognitive decline in Parkinson disease.. Nat. Rev. Neurol..

[9] Matsumoto M. (2015). Dopamine signals and physiological origin of cognitive dysfunction in Parkinson’s disease.. Mov. Disord..

[10] McGuigan S., Zhou S.H., Brosnan M.B., Thyagarajan D., Bellgrove M.A., Chong T.T.J. (2019). Dopamine restores cognitive motivation in Parkinson’s disease.. Brain.

[11] Pillon B., Czernecki V., Dubois B. (2003). Dopamine and cognitive function.. Curr. Opin. Neurol..

[12] Chaudhuri K.R., Healy D.G., Schapira A.H.V. (2006). Non-motor symptoms of Parkinson’s disease: Diagnosis and management.. Lancet Neurol..

[13] Armstrong M.J., Okun M.S. (2020). Diagnosis and treatment of Parkinson disease.. JAMA.

[14] Braak H., Tredici K.D., Rüb U., de Vos R.A.I., Jansen Steur E.N.H., Braak E. (2003). Staging of brain pathology related to sporadic Parkinson’s disease.. Neurobiol. Aging.

[15] Buongiorno M., Compta Y., Martí M.J. (2011). Amyloid-β and τ biomarkers in Parkinson’s disease–dementia.. J. Neurol. Sci..

[16] Siepel F.J., Brønnick K.S., Booij J., Ravina B.M., Lebedev A.V., Pereira J.B., Grüner R., Aarsland D. (2014). Cognitive executive impairment and dopaminergic deficits in de novo Parkinson’s disease.. Mov. Disord..

[17] Bohnen N.I., Albin R.L. (2011). The cholinergic system and Parkinson disease.. Behav. Brain Res..

[18] Liu A.K.L., Chang R.C.C., Pearce R.K.B., Gentleman S.M. (2015). Nucleus basalis of Meynert revisited: Anatomy, history and differential involvement in Alzheimer’s and Parkinson’s disease.. Acta Neuropathol..

[19] Bocanegra García Y., Trujillo Orrego N., Pineda Salazar D.A. (2014). Demencia y deterioro cognitivo leve en la enfermedad de Parkinson: Una revisión.. Rev. Neurol..

[20] Holland N., Robbins T.W., Rowe J.B. (2021). The role of noradrenaline in cognition and cognitive disorders.. Brain.

[21] Riekkinen M., Kejonen K., Jäkälä P., Soininen H., Riekkinen P. (1998). Reduction of noradrenaline impairs attention and dopamine depletion slows responses in Parkinson’s disease.. Eur. J. Neurosci..

[22] Ballanger B., Strafella A.P., van Eimeren T., Zurowski M., Rusjan P.M., Houle S., Fox S.H. (2010). Serotonin 2A receptors and visual hallucinations in Parkinson disease.. Arch. Neurol..

[23] Fox S.H., Chuang R., Brotchie J.M. (2009). Serotonin and Parkinson’s disease: On movement, mood, and madness.. Mov. Disord..

[24] Cacabelos R., Takeda M., Winblad B. (1999). The glutamatergic system and neurodegeneration in dementia: Preventive strategies in Alzheimer’s disease.. Int. J. Geriatr. Psychiatry.

[25] Wesnes K.A., Aarsland D., Ballard C., Londos E. (2015). Memantine improves attention and episodic memory in Parkinson’s disease dementia and dementia with Lewy bodies.. Int. J. Geriatr. Psychiatry.

[26] Bandini F., Pierantozzi M., Bodis-Wollner I. (2002). The visuo-cognitive and motor effect of amantadine in non-Caucasian patients with Parkinson’s disease. A clinical and electrophysiological study.. J. Neural Transm. (Vienna).

[27] Fox S.H., Katzenschlager R., Lim S.Y., Barton B., de Bie R.M.A., Seppi K., Coelho M., Sampaio C. (2018). International Parkinson and movement disorder society evidence-based medicine review: Update on treatments for the motor symptoms of Parkinson’s disease.. Mov. Disord..

[28] Widnell K. (2005). Pathophysiology of motor fluctuations in Parkinson’s disease.. Mov. Disord..

[29] Cilia R., Cereda E., Akpalu A., Sarfo F.S., Cham M., Laryea R., Obese V., Oppon K., Del Sorbo F., Bonvegna S., Zecchinelli A.L., Pezzoli G. (2020). Natural history of motor symptoms in Parkinson’s disease and the long-duration response to levodopa.. Brain.

[30] Espay A.J., Morgante F., Merola A., Fasano A., Marsili L., Fox S.H., Bezard E., Picconi B., Calabresi P., Lang A.E. (2018). Levodopa-induced dyskinesia in Parkinson disease: Current and evolving concepts.. Ann. Neurol..

[31] Wirdefeldt K., Odin P., Nyholm D. (2016). Levodopa–carbidopa intestinal gel in patients with Parkinson’s disease: A systematic review.. CNS Drugs.

[32] Jenner P., Katzenschlager R. (2016). Apomorphine - pharmacological properties and clinical trials in Parkinson’s disease.. Parkinsonism Relat. Disord..

[33] Parkinson Study Group (1989). DATATOP: A multicenter controlled clinical trial in early Parkinson’s disease.. Arch. Neurol..

[34] Sivertsen B., Dupont E., Mikkelsen B., Mogensen P., Rasmussen C., Boesen F., Heinonen E. (1989). Selegiline and levodopa in early or moderately advanced Parkinson’s disease: A double-blind controlled short- and long-term study.. Acta Neurol. Scand..

[35] Parkinson Study Group (2005). A randomized placebo-controlled trial of rasagiline in levodopa-treated patients with Parkinson disease and motor fluctuations: The PRESTO study.. Arch. Neurol..

[36] Elmer L.W. (2013). Rasagiline adjunct therapy in patients with Parkinson’s disease: Post hoc analyses of the PRESTO and LARGO trials.. Parkinsonism Relat. Disord..

[37] Hattori N., Takeda A., Takeda S., Nishimura A., Kato M., Mochizuki H., Nagai M., Takahashi R. (2018). Efficacy and safety of adjunctive rasagiline in Japanese Parkinson’s disease patients with wearing-off phenomena: A phase 2/3, randomized, double-blind, placebo-controlled, multicenter study.. Parkinsonism Relat. Disord..

[38] Schapira A.H.V., Fox S.H., Hauser R.A., Jankovic J., Jost W.H., Kenney C., Kulisevsky J., Pahwa R., Poewe W., Anand R. (2017). Assessment of safety and efficacy of safinamide as a levodopa adjunct in patients with Parkinson disease and motor fluctuations.. JAMA Neurol..

[39] Borgohain R., Szasz J., Stanzione P., Meshram C., Bhatt M., Chirilineau D., Stocchi F., Lucini V., Giuliani R., Forrest E., Rice P., Anand R. (2014). Randomized trial of safinamide add‐on to levodopa in Parkinson’s disease with motor fluctuations.. Mov. Disord..

[40] Hattori N., Tsuboi Y., Yamamoto A., Sasagawa Y., Nomoto M. (2020). Efficacy and safety of safinamide as an add-on therapy to L-DOPA for patients with Parkinson’s disease: A randomized, double-blind, placebo-controlled, phase II/III study.. Parkinsonism Relat. Disord..

[41] Gray R., Patel S., Ives N., Rick C., Woolley R., Muzerengi S., Gray A., Jenkinson C., McIntosh E., Wheatley K., Williams A., Clarke C.E., Young K., Price H., Price J., Lambert A., Reeve R., Sewell M., Broome S., Williams A., Baker M., Clarke C., Fitzpatrick R., Gray A., Greenhall R., Jenkinson C., Mant D., McIntosh E., Sandercock P., Baugent C., Crome P., Au P., Boodell T., Cheed V.C., Daniels J., Dowling F., Evans L., Hawker R., Kaur S., Rick C., Wheatley K., Winkles N., Hingley D., Sturdy L., Wooley R., Ottridge R., Peto L., Hilken N., Counsell C., Caie L., Caslake R., Coleman R., Crowley P., Gerrie L., Gordon J., Harris C., Leslie V., MacLeod M.A., Taylor K., Worth P., Barker R.A., Forsyth D., Halls M., Young J., Phillips W., Manford M., Thangarajah N., Blake D., Prescott R., Carr P., Cochrane L., Rose A., McLaren A., Drover M., Karunaratne P., Eady A., Wislocka-Kryjak M., Ghaus N., Grueger A., Mallinson B., Wihl G., Ballantyne S., Hutchinson S., Lewthwaite A., Nicholl D., Ritch A., Coyle S., Hornabrook R., Irfan H., Poxon S., Nath U., Davison J., Dodds S., Robinson G., Gray C., Fletcher P., Morrow P., Sliva M., Folkes E., Gilbert A., Hayes H., Burrows E., Donaldson S., Lawrence J., Rhind G., Baxter G., Bell J., Gorman J., Guptha S., Noble C., Hindle J., Jones S., Ohri P., Subashchandran R., Roberts E., Raw J., Wadhwa U., Aspden L., Partington L., Vanek H., Whone A., Barber R., Haywood B., Heywood P., Lewis H., O’Sullivan K., Prout K., Whelan L., Medcalf P., Sliva M., Fuller G., Morrish P., Wales E., Dalziel J., Overstall P., Bouifraden K., Evans C., Ward G., Matheson P., Lockington T., Graham A., Grimmer S.F.M., Sheehan L.J., Williams H., Hubbard I., Walters R., Glasspool R., Critchley P., Abbott R., Kendall B., Lawden M., Lo N., Rajaally Y., Simpson B., Martey J., Wray L.G., Omar M., Sharma A., Gale A., Phirii D., Sekaran L., Wijayasiri S., Silverdale M., Walker D., Fleary H., Monaghan A., Senthil V., Reynolds S., Chong M.S., Diem D., Kundu B., Arnold D., Quinn N., Benamer H., Billings J., Corston R., D’Costa D., Green M., Shuri J., Noble J.M., Cassidy T., Gani A., Lawson R., Nirubin A., Cochius J., Dick D., Lee M., Payne B., Roche M., Sabanathan K., Shields S., Hipperson M., Reading F., Saunders J., Harper G., Honan W., Gill L., Stanley J., Vernon N., Skinner A., McCann P., Walker R., Edmonds P., O’Hanlon S., Wood B., Hand A., Robinson L., Liddle J., Bolam D., Raha S., Ebebezer L., Thompson S., Pall H., Praamstra P., Crouch R., Healy K., Johnson M., Jenkinson M., Abdel-Hafiz A., Al-Modaris F., Dutta S., Mallik T., Mondal B., Roberts J., Sinha S., Amar K., Atkins S., Devadason G., Martin A., Cox C., Malone T., Fenwick G., Gormley K., Gutowski N., Harris S., Harrower T., Hemsley A., James M., Jeffreys M.O., Pearce V., Sheridan R., Sword J., Zeman A., Soper C., Vassallo J., Bennett J., Lyell V., Robertson D., Howcroft D., Mugweni K., Stephens A., Whelan E., Wright A., Chamberlain J., Padiachy D., Marigold J., Lee J., Roberts H., Adams J., Dulay J., Evans S., Frankel J., Gove R., Turner G., Mallik N., McElwaine T., Morgan S., Phipps H., Pressly V., Queen V., Tan R., Grossett D., Macphee G., Vennard C., Rektorova I., Dhakam Z., Carey G., Castledon B., Sunderland C., Kalcantera E., Long C., Mandal B., Martin V., Nari R., Nicholas V., Moffitt V., Hammans S., Rice-Oxley M., Webb J., Franks S., Cooper S., Hussain M., Solanki T., Darch W., Homan J., Sharratt D., Griggs G., Kendall G., Ford A., Stocker K., Strens L., Grubneac A., Ponsford J., Teare L., Moore A.P., O’Brien I., Watling D., Wyatt L., Rizvi S., Walker E., Berry G., Russell N., Rashed K., Baker K., Qadiri M.R., Buckley C., Bulley S., Gibbons D., Goodland R., Heywood P., Jones L., Martin L., Rowland-Axe R., Stone A., Whittuck M.R. (2022). Long-term effectiveness of adjuvant treatment with catechol-O-methyltransferase or monoamine oxidase B inhibitors compared with dopamine agonists among patients with Parkinson disease uncontrolled by levodopa therapy.. JAMA Neurol..

[42] Csanda E., Tárczy M. (1987). Selegiline in the early and late phases of Parkinson’s disease.. J. Neural Transm. Suppl..

[43] Parkinson Study Group (2002). A controlled trial of rasagiline in early Parkinson disease: The TEMPO Study.. Arch. Neurol..

[44] Jankovic J., Berkovich E., Eyal E., Tolosa E. (2014). Symptomatic efficacy of rasagiline monotherapy in early Parkinson’s disease: Post-hoc analyses from the ADAGIO trial.. Parkinsonism Relat. Disord..

[45] Magyar K. (2011). The pharmacology of selegiline.. Int. Rev. Neurobiol..

[46] Alborghetti M., Nicoletti F. (2019). Different generations of type-B monoamine oxidase inhibitors in Parkinson’s disease: From bench to bedside.. Curr. Neuropharmacol..

[47] Hietanen M.H. (1991). Selegiline and cognitive function in Parkinson’s disease.. Acta Neurol. Scand..

[48] Dalrymple-Alford J.C., Jamieson C.F., Donaldson I.M. (1995). Effects of selegiline (deprenyl) on cognition in early Parkinson’s disease.. Clin. Neuropharmacol..

[49] Murakami H., Momma Y., Nohara T., Mori Y., Futamura A., Sugita T., Ishigaki S., Katoh H., Kezuka M., Ono K., Miller M.W., Kawamura M. (2016). Improvement in language function correlates with gait improvement in drug-naïve Parkinson’s disease patients taking dopaminergic medication.. J. Parkinsons Dis..

[50] Portin R., Rinne U.K. (1983). The effect of deprenyl (selegiline) on cognition and emotion in parkinsonian patients undergoing long-term levodopa treatment.. Acta Neurol. Scand..

[51] Barone P., Santangelo G., Morgante L., Onofrj M., Meco G., Abbruzzese G., Bonuccelli U., Cossu G., Pezzoli G., Stanzione P., Lopiano L., Antonini A., Tinazzi M. (2015). A randomized clinical trial to evaluate the effects of rasagiline on depressive symptoms in non‐demented Parkinson’s disease patients.. Eur. J. Neurol..

[52] Hanagasi H.A., Gurvit H., Unsalan P., Horozoglu H., Tuncer N., Feyzioglu A., Gunal D.I., Yener G.G., Cakmur R., Sahin H.A., Emre M. (2011). The effects of rasagiline on cognitive deficits in Parkinson’s disease patients without dementia: A randomized, double-blind, placebo-controlled, multicenter study.. Mov. Disord..

[53] Rinaldi D., Assogna F., Sforza M., Tagliente S., Pontieri F.E. (2018). Rasagiline for dysexecutive symptoms during wearing-off in Parkinson’s disease: A pilot study.. Neurol. Sci..

[54] Weintraub D., Hauser R.A., Elm J.J., Pagan F., Davis M.D., Choudhry A. (2016). Rasagiline for mild cognitive impairment in Parkinson’s disease: A placebo-controlled trial.. Mov. Disord..

[55] Frakey L.L., Friedman J.H. (2017). Cognitive effects of rasagiline in mild-to-moderate stage Parkinson’s disease without dementia.. J. Neuropsychiatry Clin. Neurosci..

[56] Salvati P., Maj R., Caccia C., Cervini M.A., Fornaretto M.G., Lamberti E., Pevarello P., Skeen G.A., White H.S., Wolf H.H., Faravelli L., Mazzanti M., Mancinelli E., Varasi M., Fariello R.G. (1999). Biochemical and electrophysiological studies on the mechanism of action of PNU-151774E, a novel antiepileptic compound.. J. Pharmacol. Exp. Ther..

[57] Morari M., Brugnoli A., Pisanò C.A., Novello S., Caccia C., Melloni E., Padoani G., Vailati S., Sardina M. (2018). Safinamide differentially modulates in vivo glutamate and GABA release in the rat hippocampus and basal ganglia.. J. Pharmacol. Exp. Ther..

[58] Onofrj M., Bonanni L., Thomas A. (2008). An expert opinion on safinamide in Parkinson’s disease.. Expert Opin. Investig. Drugs.

[59] Conn P.J., Battaglia G., Marino M.J., Nicoletti F. (2005). Metabotropic glutamate receptors in the basal ganglia motor circuit.. Nat. Rev. Neurosci..

[60] Cattaneo C., Sardina M., Bonizzoni E. (2016). Safinamide as add-on therapy to levodopa in mid- to late-stage Parkinson’s disease fluctuating patients: Post hoc analysesof studies 016 and SETTLE.. J. Parkinsons Dis..

[61] Borgohain R., Szasz J., Stanzione P., Meshram C., Bhatt M.H., Chirilineau D., Stocchi F., Lucini V., Giuliani R., Forrest E., Rice P., Anand R. (2014). Two‐year, randomized, controlled study of safinamide as add‐on to levodopa in mid to late Parkinson’s disease.. Mov. Disord..

[62] Abbruzzese G., Kulisevsky J., Bergmans B., Gomez-Esteban J.C., Kägi G., Raw J., Stefani A., Warnecke T., Jost W.H. (2021). A European observational study to evaluate the safety and the effectiveness of safinamide in routine clinical practice: The SYNAPSES Trial1.. J. Parkinsons Dis..

[63] Cattaneo C., Müller T., Bonizzoni E., Lazzeri G., Kottakis I., Keywood C. (2017). Long-term effects of safinamide on mood fluctuations in Parkinson’s disease.. J. Parkinsons Dis..

[64] Cattaneo C., Barone P., Bonizzoni E., Sardina M. (2017). Effects of safinamide on pain in fluctuating Parkinson’s disease patients: A post-hoc analysis.. J. Parkinsons Dis..

[65] Cattaneo C., Kulisevsky J., Tubazio V., Castellani P. (2018). Long-term efficacy of safinamide on Parkinson’s disease chronic pain.. Adv. Ther..

[66] Liguori C., Mercuri N.B., Stefani A., Pierantozzi M. (2018). Effective treatment of restless legs syndrome by safinamide in Parkinson’s disease patients.. Sleep Med..

[67] Bianchi M.L.E., Riboldazzi G., Mauri M., Versino M. (2019). Efficacy of safinamide on non-motor symptoms in a cohort of patients affected by idiopathic Parkinson’s disease.. Neurol. Sci..

[68] De Micco R., Satolli S., Siciliano M., De Mase A., Giordano A., Tedeschi G., Tessitore A. (2022). Effects of safinamide on non-motor, cognitive, and behavioral symptoms in fluctuating Parkinson’s disease patients: A prospective longitudinal study.. Neurol. Sci..

[69] Rinaldi D., Sforza M., Assogna F., Savini C., Salvetti M., Caltagirone C., Spalletta G., Pontieri F.E. (2021). Safinamide improves executive functions in fluctuating Parkinson’s disease patients: An exploratory study.. J. Neural Transm. (Vienna).

[70] Bundgaard C., Montezinho L.P., Anderson N., Thomsen C., Mørk A. (2016). Selegiline induces a wake promoting effect in rats which is related to formation of its active metabolites.. Pharmacol. Biochem. Behav..

[71] Yasar S., Goldberg J.P., Goldberg S.R. (1996). Are metabolites of l-deprenyl (selegiline) useful or harmful? Indications from preclinical research.. J. Neural Transm. Suppl..

[72] Biundo R., Weis L., Antonini A. (2016). Cognitive decline in Parkinson’s disease: The complex picture.. NPJ Parkinsons Dis..

[73] Narayanan N.S., Rodnitzky R.L., Uc E.Y. (2013). Prefrontal dopamine signaling and cognitive symptoms of Parkinson’s disease.. Rev. Neurosci..

[74] Borovac J.A. (2016). Side effects of a dopamine agonist therapy for Parkinson’s disease: A mini-review of clinical pharmacology.. Yale J. Biol. Med..

[75] Fenu S., Wardas J., Morelli M. (2009). Impulse control disorders and dopamine dysregulation syndrome associated with dopamine agonist therapy in Parkinson’s disease.. Behav. Pharmacol..

[76] Martinez-Martin P., Wan Y.M., Ray Chaudhuri K., Schrag A.E., Weintraub D. (2020). Impulse control and related behaviors in Parkinson’s disease with dementia.. Eur. J. Neurol..

[77] Anderson K.E. (2004). Behavioral disturbances in Parkinson’s disease.. Dialogues Clin. Neurosci..

[78] Trojano L., Papagno C. (2018). Cognitive and behavioral disorders in Parkinson’s disease: An update. II: Behavioral disorders.. Neurol. Sci..

[79] Merims D., Giladi N. (2008). Dopamine dysregulation syndrome, addiction and behavioral changes in Parkinson’s disease.. Parkinsonism Relat. Disord..

[80] Antonelli F., Strafella A.P. (2014). Behavioral disorders in Parkinson’s disease: The role of dopamine.. Parkinsonism Relat. Disord..

[81] Nyholm D., Jost W.H. (2021). An updated calculator for determining levodopa-equivalent dose.. Neurol. Res. Pract..

[82] Verber D., Novak D., Borovič M., Dugonik J., Flisar D. (2020). EQUIDopa: A responsive web application for the levodopa equivalent dose calculator.. Comput. Methods Programs Biomed..

[83] Schade S., Mollenhauer B., Trenkwalder C. (2020). Levodopa equivalent dose conversion factors: An updated proposal including opicapone and safinamide.. Mov. Disord. Clin. Pract. (Hoboken).

[84] Geroin C., Di Vico I.A., Squintani G., Segatti A., Bovi T., Tinazzi M. (2020). Effects of safinamide on pain in Parkinson’s disease with motor fluctuations: An exploratory study.. J. Neural Transm. (Vienna).

[85] Gómez-López A., Sánchez-Sánchez A., Natera-Villalba E., Ros-Castelló V., Beltrán-Corbellini Á., Fanjul-Arbós S., Pareés M.I., López-Sendon Moreno J.L., Martínez Castrillo J.C., Alonso-Canovas A. (2021). SURINPARK: Safinamide for Urinary Symptoms in Parkinson’s Disease.. Brain Sci..

[86] Gardoni F., Morari M., Kulisevsky J., Brugnoli A., Novello S., Pisanò C.A., Caccia C., Mellone M., Melloni E., Padoani G., Sosti V., Vailati S., Keywood C. (2018). Safinamide modulates striatal glutamatergic signaling in a rat model of levodopa-induced dyskinesia.. J. Pharmacol. Exp. Ther..

[87] Morin N., Morissette M., Grégoire L., Di Paolo T. (2016). mGlu5, Dopamine D2 and Adenosine A2A Receptors in L-DOPA-induced Dyskinesias.. Curr. Neuropharmacol..

[88] Litim N., Morissette M., Di Paolo T. (2017). Metabotropic glutamate receptors as therapeutic targets in Parkinson’s disease: An update from the last 5 years of research.. Neuropharmacology.

[89] Calabresi P., Giacomini P., Centonze D., Bernardi G. (2000). Levodopa-induced dyskinesia: A pathological form of striatal synaptic plasticity?. Ann. Neurol..

[90] Tozzi A., Sciaccaluga M., Loffredo V., Megaro A., Ledonne A., Cardinale A., Federici M., Bellingacci L., Paciotti S., Ferrari E., La Rocca A., Martini A., Mercuri N.B., Gardoni F., Picconi B., Ghiglieri V., De Leonibus E., Calabresi P. (2021). Dopamine-dependent early synaptic and motor dysfunctions induced by α-synuclein in the nigrostriatal circuit.. Brain.

[91] Guerra A., Suppa A., D’Onofrio V., Di Stasio F., Asci F., Fabbrini G., Berardelli A. (2019). Abnormal cortical facilitation and L-dopa-induced dyskinesia in Parkinson’s disease.. Brain Stimul..

[92] Guerra A., Asci F., Zampogna A., D’Onofrio V., Suppa A., Fabbrini G., Berardelli A. (2022). Long-term changes in short-interval intracortical facilitation modulate motor cortex plasticity and L-dopa-induced dyskinesia in Parkinson’s disease.. Brain Stimul..

